# Functional divergence of gene duplicates – a domain-centric view

**DOI:** 10.1186/1471-2148-12-126

**Published:** 2012-07-27

**Authors:** Mugdha Khaladkar, Sridhar Hannenhalli

**Affiliations:** 1 Department of Biology, University of Pennsylvania, Philadelphia, PA, 19104, USA; 2 University of Maryland, 3104G Biomolecular Sciences Building (#296), College Park, MD, USA

**Keywords:** Gene duplication, Whole genome duplication, Computational biology, Evolution

## Abstract

**Background:**

Gene duplicates have been shown to evolve at different rates. Here we further investigate the mechanism and functional underpinning of this phenomenon by assessing asymmetric evolution specifically within functional domains of gene duplicates.

**Results:**

Based on duplicate genes in five teleost fishes resulting from a whole genome duplication event, we first show that a Fisher Exact test based approach to detect asymmetry is more sensitive than the previously used Likelihood Ratio test. Using our Fisher Exact test, we found that the evolutionary rate asymmetry in the overall protein is largely explained by the asymmetric evolution within specific protein domains. Moreover, among cases of asymmetrically evolving domains, for the gene copy containing a fast evolving domain, the non-synonymous substitutions often cluster within the fast evolving domain. We found that rare substitutions were preferred within asymmetrically evolving domains suggestive of functional divergence. While overall ~32 % of the domains tested were found to be evolving asymmetrically, certain protein domains such as the Tyrosine and Ser/Thr Kinase domains had a much greater prevalence of asymmetric evolution. Finally, based on the spatial expression of Zebra fish duplicate proteins during development, we found that protein pairs containing asymmetrically evolving domains had a greater divergence in gene expression as compared to the duplicate proteins that did not exhibit asymmetric evolution.

**Conclusions:**

Taken together, our results suggest that the previously observed asymmetry in the overall duplicate protein evolution is largely due to divergence of specific domains of the protein, and coincides with divergence in spatial expression domains.

## Background

Gene duplication plays a critical role in driving evolutionary diversity by supplying large, functional, and redundant DNA sequences as the raw material for evolution. In proposing a model to describe the possible fates of duplicated genes, Ohno postulated that the redundancy created by duplication results in a reduced selective constraint that allows a redundant locus to accumulate mutations, which may either result in its *non-functionalization* or lead to the development of a hitherto new functionality termed as *neofunctionalization (NF)*[1]. A third possibility that was not considered in Ohno’s model arises when both the copies undergo mutually complementary degenerative mutations leading to *subfunctionalization (SF)* of the ancestral function [2]. A duplicated locus that undergoes *NF* is expected to evolve faster than the paralogous locus, and thus an evolutionary analysis of the two loci should reveal an asymmetry in their rates of molecular evolution. In the case of *SF* asymmetric evolution may not be apparent at the whole-protein level because the two copies would include a different fast evolving functional domain. However, an increased evolutionary rate may be due to a relaxed purifying selection or a positive selection on a few residues; these two possibilities cannot always be distinguished based on evolutionary rate alone.

Asymmetric sequence evolution of gene duplicates has been observed in several prior studies. Conant and Wagner found 20-30 % of duplicated gene pairs to be evolving asymmetrically in yeast, fly and nematode [3]. Byrne and Wolf found 56 % of the duplicates in three yeast species to be evolving asymmetrically [4]. Another study that analyzed gene duplicates arising from whole genome duplication (WGD) from three teleost fish genomes reported asymmetric evolution in at least 36 % of the cases [5]. However, in all previous studies, the rate of asymmetry was analyzed on the whole protein level. Proteins have a modular structure composed of evolutionary conserved functional units, termed *domains* which contribute to various catalytic and interaction functions. The domains are intervened by variable non-domain regions which largely contribute to the structure of the protein [6]. Because of modular nature of proteins, analysis of asymmetric evolution and functional divergence of gene duplicates at the level of functional domains offers several advantages over analysis at the level of the entire protein sequence. First, if the asymmetric evolution is limited to a small domain, it may remain undetected due to low signal to noise ratio. Second, if different domains are fast evolving in the two gene copies, as possibly in the case of *SF*, they may remain undetected at the overall protein level as the opposing signals of asymmetry within these domains would cancel out. Third, a domain-centric analysis of asymmetry uniquely enables biological interpretation via functional investigation of the asymmetrically evolving domains.

Here we report a large scale analysis of asymmetric evolution of gene duplicates in five teleost fish genomes [7]. These duplicates were a result of a teleost specific WGD event ~305-450 MYA [8,9] and thus have evolved over an identical evolutionary time, providing a suitable dataset for a large scale study. We first assessed whole protein level asymmetric evolution among 605 duplicate pairs in the five species using a Fisher Exact test based approach that we found to be more sensitive than the previously used Likelihood Ratio test. We thus detected asymmetric evolution in 50-65 % (at 10 % FDR) of the gene pairs, greater than what has been previously reported. We further assessed asymmetric evolution in the annotated protein domains and found that a large fraction of the asymmetry detected at the whole protein level can be attributed to a specific domain. Moreover, several cases of asymmetric evolution were uniquely detected in the domain-centric analysis, despite reduced statistical power, likely due to a better signal to noise ratio. Our finding that the asymmetry in evolutionary rate is primarily localized within specific domains is further supported by the result that non-synonymous changes between the two duplicates are clustered within few of its domains and not spread evenly across all the domains. More importantly, as should be expected, we found that most of the domains that showed a signal for asymmetry also showed clustering of non-synonymous changes within them. We also found that asymmetrically evolving domains are targeted for qualitatively different substitutions as compared to symmetrically evolving domains, consistent with a functional divergence between duplicated genes. Interestingly, for gene duplicate pairs with multiple asymmetrically evolving domains, the faster evolving domain copies were from one of the paralog of the duplicate pair in most cases; this is consistent with *NF* as the prevalent mechanism underlying diversification of the gene duplicates, also suggested by some of the previous studies [3-5]. Lastly, based on ZFIN database, a comprehensive database of *Danio rerio* spatio-temporal gene expression [10], we found that the protein pairs with asymmetrically evolving domains showed significantly greater divergence in gene expression as compared to the protein pairs with no asymmetrically evolving domain. Overall, our study provides novel insights into the divergence of gene duplicates by focusing on asymmetric evolution of individual functional domains of the protein.

## Results and discussion

### Widespread asymmetry in the rate of evolution

For each pair of paralogous fish genes and the corresponding mouse ortholog used as an outgroup, we first assessed, using the likelihood-ratio test (LRT) (see Methods), whether unconstrained *ω* on the two fish lineages explains the data significantly better than the constrained model where both fish lineages have an identical *ω*. Using a FDR threshold of 10 %, LRT supported asymmetry in <10 % of the duplicate pairs for each fish species (Table 1). This fraction of asymmetrically evolving gene duplicates is lower relative to previous similar studies. Conant and Wagner found 20-30 % paralogous gene pairs to be asymmetric evolving in yeast, fly and nematode by selecting an appropriate outgroup gene for each duplicate pair from within the same species [3]. Byrne and Wolfe analyzed WGD duplicates in three yeast species by comparing to a close pre-WGD yeast species as the outgroup and found 56 % of the pairs to be evolving asymmetrically [4]. Another study that used three of the teleost fish gene duplicates reported asymmetric evolution in at least 36 % of the gene pairs by comparing the differences in dN to the differences in dS between the paralogs while using human-mouse orthologous pair as the comparison group [5]. One reason for the much reduced detection for asymmetry in our study may be that the evolutionary time since duplication is much shorter than the time since the common ancestor of fish and mouse. Indeed, the estimate of dS for the fish-mouse branch was well above 2 for almost all genes used in this analysis. Therefore, this shared sequence variation between the two models can dominate the likelihood terms, which would result in lack of any significant difference between the likelihoods of the two models. Unfortunately, comprehensive gene sequence data for a more closely related outgroup species are not available. None of the previous studies suffer from this shortcoming either because of choice of species [3,4] or because of an indirect comparison of fish paralogs dN and dS difference with that of human-mouse orthologs [5], which presumes a preservation of function among the human-mouse orthologs.

**Table 1:** Number and percentage of paralogs deemed to be asymmetrically evolving (FDR = 10 %) based on the whole protein sequence and using two different methods – Likelihood ratio test (LRT), and Fisher Exact test (FET)

**Species**	**Asymmetry (LRT)**	**Asymmetry (FET)**
*D. rerio*	7/119 (5.8 %)	77/119 (64.7 %)
*O. latipes*	13/144 (9.1 %)	86/144 (59.7 %)
*G. aculeatus*	12/159 (7.5 %)	80/159 (50.3 %)
*T. nigrovirdis*	5/64 (7.8 %)	36/64 (56.2 %)
*T. rubripes*	8/119 (6.7 %)	69/119 (57.9 %)

To specifically evaluate the difference between the two branches leading to the two fish duplicates, we devised the FET approach (see Methods) that directly assesses the difference in non-synonymous changes in the two branches using synonymous changes as the comparison group. FET has been previously used to test for neutral evolution along a branch by comparing the ratio of non-synonymous to synonymous substitutions along the specific branch to the ratio of overall non-synonymous to overall synonymous sites in the sequence [11]. Interestingly, FET detected 50-65 % (at 10 % FDR) of the gene duplicates as evolving asymmetrically (Table 1). These results hold even at a more stringent 5 % FDR (Additional file 1: Table S1). These asymmetric fractions are greater than the fractions reported previously for teleost fish, yeast, nematode and fly [3-5]. Even at a much more stringent FDR of 0.01 % FET detected 22-28 % asymmetry which is still much greater than the asymmetry detected using LRT (Additional file 1: Table S2). Lending support to our conjecture that the lower power of LRT was due to the larger shared evolution between the two models was the finding that upon including the shared branches in the FET analysis there was on average ~35 % reduction in the asymmetric signal for each of the five fish gene duplicates. However, it is possible that the greater asymmetry detected by FET may be due to a higher false positive rate for the FET approach than that for the LRT approach. Therefore, we carefully assessed the false discovery rate as well as the robustness of the FET approach.

**Figure 1:**
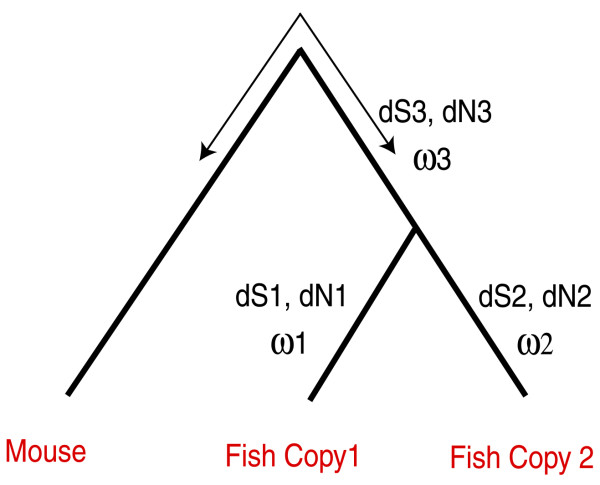
**Un-rooted tree topology for the fish gene duplicates and the mouse orthologous gene used as outgroup.** Evolutionary rates are obtained for every branch of this tree.

First, we assessed the false positive rate of the FET approach via simulation using the *evolver* program of the PAML package [12] for the evolutionary scenario depicted in Figure 1 under conditions of neutral evolution with all branches evolving at the same rate (see Methods). The branch lengths for the tree used for simulated evolution was derived from the same underlying data to ensure that the total evolution was comparable between the actual data and the simulated data. FET approach reported significant asymmetry in only 2 % of the instances (P-value < = 0.05 at 10 % FDR).

Secondly, since PAML does not report error estimates for the predicted dS and dN values and both FET and LRT analyses treat these estimates as is, which may adversely affect the robustness of the inference of asymmetry. Therefore we assessed the robustness of the FET approach using bootstrapping. For all asymmetrically evolving protein duplicates, we sampled codons randomly with replacement from the aligned proteins to form randomized sequences of the same original length, and repeated the FET analysis on 100 such randomized set. All asymmetrically evolving proteins manifested significant asymmetry in 80 % of the randomized cases on average (FDR = 10 %). At the much higher stringent FDR of 0.01 %, the bootstrapping support increased to ~90 %. Thus, the FET analysis for detection of asymmetry is relatively sensitive and robust. As a compromise between maintaining a low false positive rate while still detecting enough cases of asymmetry for meaningful analysis, we chose to use FDR of 10 % for all further analysis.

In addition, we found that the non-synonymous substitutions in each of the two fish gene copies relative to the mouse ortholog occur in different, non-interleaved, regions of the protein. In other words, the positions of the ancestral protein that is uniquely mutated in exactly one of the gene copies, tend to form contiguous runs and do not interleave with each other (P-value = 3.4e-15; Additional file 1: Supplementary Result). Assuming that different regions of the protein encode different aspects of the protein functionality, this finding suggests that sequence divergence of the two copies tends to target specific aspects of the ancestral function.

### Signature of asymmetric evolution is largely contained within the protein domains

Next, we analyzed whether the widespread asymmetry detected above predominantly arises from the functional domain of the proteins, and whether more instances of asymmetric evolution may be detected by focusing exclusively on sequence variation in domains as opposed to the entire protein sequence. To do so, we excluded the regions of each protein that was not annotated as a Pfam domain (see Methods). This procedure resulted in 57 % reduction in sequence length on average. We repeated FET based analysis of asymmetry on the reduced sequences, which is a concatenation of all annotated domains. We refer to this analysis as the *Combined Domain Analysis* (*CDA*). This is contrasted with analysis above, based on the whole proteins sequences, referred to as *Whole Protein Analysis* (*WPA*). To elucidate our findings, here we only discuss the results for multi-domain proteins. As shown in Table 2, relative to WPA, the CDA detects slightly fewer cases of asymmetric evolution (12.7 % reduction on average). However, we reasoned that this may be due to loss of power from reduced sequence lengths rather than reduced asymmetric evolution. We confirmed this conjecture based on random sampling of multiple alignment columns in the WPA so as to match the alignment lengths in the CDA, and as suspected, on average, the sampled WPA reduces the number of cases of asymmetric evolution as compared to WPA and yields a comparable fraction of asymmetric evolution as that for the CDA (Additional file 1: Table S3).

**Table 2:** **Fisher Exact test based analysis of asymmetry in three different settings – Whole Protein (WPA), Combined Domain (CDA), and Domain Specific (DSA).** The table shows the total pairs tested and fraction deemed to be asymmetrically evolving (FDR = 10 %)

**Species**	**WPA**	**CDA**	**DSA**	
			**wrt proteins**	**wrt domains**
*D. rerio*	32/45 (71.1 %)	25/45 (55.5 %)	28/45 (62.2 %)	41/134 (30.6 %)
*O. latipes*	39/67 (58.2 %)	29/67 (43.3 %)	37/67 (55.2 %)	55/209 (26.3 %)
*G. aculeatus*	31/68 (45.5 %)	25/68 (36.7 %)	22/68 (32.3 %)	26/209 (12.4 %)
*T. nigrovirdis*	13/25 (52.0 %)	11/25 (44.0 %)	14/25 (56.0 %)	16/67 (23.9 %)
*T. rubripes*	28/54 (51.8 %)	19/54 (35.2 %)	19/54 (35.2 %)	23/148 (15.5 %)

**Figure 2:**
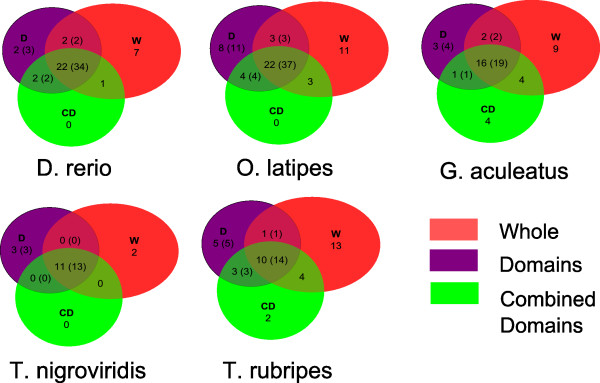
**Overlap between the asymmetrically evolving protein duplicates detected by WPA, CDA and DSA.** The numbers of protein duplicates deemed to evolve asymmetrically by each of the three analyses are indicated in the Venn diagrams. The numbers inside the paranthesis are specific to DSA and they are the number of domains from the protein duplicates that were found evolving asymmetrically.

As shown in Figure 2, the WPA detects several cases of asymmetric evolution that are missed by CDA. This may be due in part to greater statistical power, as argued above, or alternatively, due to un-annotated domains or asymmetry in evolutionary rates in the between-domain linker regions. To further probe into this possibility we tested for asymmetry using only the non-domain linker regions of the proteins. Although the fraction of asymmetry in non-domain regions was comparable to that for WPA (Additional file 1: Table S4), the overlap between the two was on average only ~65 %, much lower than that between CDA and WPA. Furthermore, the evolutionary rate in the linker regions is much greater in the linker regions compared with the whole protein owing to lower functional constraint and when using the correspondingly greater evolutionary rate in our simulation, we found a significantly greater (~50 % greater) rate of false positives and moreover evolutionary rate was even higher among the linker regions deemed to be asymmetrically evolving compared with other linker regions. Taken together, these results suggest a greater false positive in detecting asymmetry in the linker regions. However, we cannot rule out the possibility that some cases of asymmetry in the linker regions may be due to the presence of un-annotated domains or their inherent functional constraints.

Taken together, our results suggest that for vast majority of the proteins evolving asymmetrically the overall signal of asymmetry is detectable when considering only the functional domains of the protein.

### Domain-specific signatures of asymmetric evolution

Protein domains provide functional modularity, and it is possible that functional divergence between duplicate genes exploits this modularity. For instance, it was previously observed that the functional divergence of transcription factor gene duplicates in terms of their DNA binding motifs (which is directly encoded by the DNA binding domain) inversely relates to their expression divergence [13], underscoring the importance of domain-centric analysis of functional divergence of paralogs. Therefore, we investigated the extent to which the overall signature of asymmetric evolution in the domains (CDA) can be localized within specific domains. We repeated the FET based analysis of asymmetry independently on each annotated domain of each gene paralog. We refer to this analysis as the *Domain Specific Analysis* (*DSA*). As shown in Table 2, at the level of individual domains, a smaller fraction of cases were detected as evolving asymmetrically. As before, based on sampling, we found that this is largely due to reduced data and thus reduced statistical power (Additional file 1: Table S3). Interestingly however, the DSA is more powerful than the CDA for proteins having at least one asymmetrically evolving domain. More specifically, we found that DSA detects a much larger fraction of proteins having an asymmetrically evolving domain than does the CDA (Figure 2). This may be either due to improved signal-to-noise in the DSA when asymmetric evolution is concentrated in a specific domain, or due to opposite asymmetry in different domains of a protein, which may go undetected in CDA. An example of opposite asymmetry is the *OL PRKAG2* (protein kinase, AMP-activated, gamma 2 non-catalytic subunit) gene duplicate pair (ENSORLG00000003934 and ENSORLG00000016783) that comprises of three *CBS* domains (named after cystathionine β-synthase). The *CBS* domains regulate the activity of associated enzymatic and transporter domains in response to binding molecules with adenosyl groups such as AMP and ATP, or s-adenosylmethionine [14]. By performing DSA on this pair we found that one of the *CBS* domain is evolving asymmetrically faster in ENSORLG00000003934 (FET P-value = 0.008, FDR < 4 %) while another of the *CBS* domains was evolving faster in ENSORLG00000016783 (FET P-value = 0.01, FDR < 6 %). Hence, the asymmetric signals in these two domains, when combined, oppose one another and remain undetected by CDA. As an example of reduced signal-to-noise in CDA, for the *OL YES1* (Yamaguchi sarcoma viral oncogene homolog 1) gene duplicate pair (ENSORLG00000017875 and ENSORLG00000011192) which comprises of three domains: *SH3_1SH2* (Src Homology 2) and *Pkinase_Tyr* (Tyrosine kinase), DSA found the *Pkinase_Tyr* domain in ENSORLG00000011192 to be evolving faster (FET P-value = 0.002, FDR < 2 %). Tyrosine kinases are a subgroup of the larger class of protein kinases and they phosphorylate proteins and serve to carry out the crucial function of signal transduction and regulation of cellular activity [15]. While *SH3_1* and *SH2* function as helper domains that aids in the protein’s interaction with other proteins [16], they possibly add to the noise in this instance and thus the signal for asymmetry is not detected by the CDA analysis.

### Assortment of asymmetrically evolving domains among proteins

Next we tested whether asymmetrically evolving domains assort among different multi-domain proteins, or alternatively, concentrated within a few multi-domain proteins. We found that on average the number of multi-domain proteins with exactly one asymmetrically evolving domain is two-fold greater than expected by random assortment. In other words, in the asymmetrically evolving proteins, there is often one specific domain contributing to the asymmetry. This suggests that the asymmetry in the rate of evolution of gene duplicates can be largely attributed to the asymmetric evolution of a specific protein domain. However, there are a few cases (total of 35; ranging from 2 to 13 in the five species) where multiple domains evolve asymmetrically.

An asymmetrically evolving domain is evolving faster in one of the gene copies; we call this copy the faster evolving copy of the domain. In a gene pair with multiple asymmetrically evolving domains, we investigated the assortment of faster evolving domain copies among the two gene copies. In other words, we assessed whether the faster evolving copies of different domains largely belong to the same gene copy or are assorted evenly between the two gene copies. Based on the few cases, we found that in most instances (83 %; ranging from 69-100 %) all the faster evolving domains belonged to the same copy (Additional file 1: Table S5). Functional divergence of preserved gene duplicates is broadly explained by two models. According to *NF* model, one of the gene copies evolves an entirely new function while the other copy retains the ancestral original function. According to *SF* model, both copies diversify, via complementary degenerative mutations, to retain disjoint subsets of the original functions [2]. The relative importance of these two mechanisms is broadly debated [17,18] and combined *SF-NF* models have been proposed. Although, evolutionary rate alone is not sufficient to make inferences of *NF* and *SF*, our finding is more consistent with *NF* of one of the duplicates, in line with previous studies [3-5].

### Non-synonymous mutations tend to cluster within a domain

Given the prevalence of asymmetric evolution of domains, as well as the fact that typically only one of the domains in the protein evolves asymmetrically, we expect the non-synonymous substitutions to be clustered within the asymmetrically evolving domain. Therefore, we assessed whether the non-synonymous changes are clustered within specific domains or are uniformly spread across all domains of a given protein. Note that, using the mouse ortholog, we can infer the non-synonymous changes independently for each fish gene duplicate and therefore this analysis can be performed independently on each copy of the duplicate pairs (see Methods). We found that most often the non-synonymous changes are clustered within a few of the domains of a protein (Table 3). Moreover, for a large fraction (64-81 %) of the faster evolving domains, non-synonymous changes are clustered in these domains (Table 3). This result lends further support to the contribution of domain-specific asymmetric evolution to the overall asymmetric evolution.

**Table 3:** Clustering of non-synonymous substitutions within domains

**Species**	**Domains with clustering**	**Faster evolving domains with clustering**
*D. rerio*	66.6 %	64.3 %
*O. latipes*	76.1 %	72.9 %
*G. aculeatus*	68.4 %	81.8 %
*T. nigrovirdis*	64 %	71.4 %
*T. rubripes*	73.1 %	73.7 %

### Analysis of asymmetrically evolving domains

In our DSA, 353 of the total 1113 protein domain pairs (~32 %) were found to evolve asymmetrically (FET P-value < = 0.05 and FDR < = 20 %). However, there is large variability among different types of domains in their tendency to evolve asymmetrically. We computed for each domain, among all instances of the domain for all five fish species, the fraction of cases it was deemed to evolve asymmetrically by the DSA (Additional file 1: Table S6). Kinase domains were found to evolve asymmetrically in a large number of cases tested: Tyrosine kinase domain (67 %), and Ser/Thr kinase domain (50 %). However due to the low overall counts for these domains in our duplicated protein set these fractions are not significantly different from random expectation based on all domains. On the other hand certain other domains showed a significantly low occurrence of asymmetric evolution. The homeobox domain, which binds to DNA/RNA and is commonly found in transcription factors [19], occurred a total of 19 times in our dataset. However, the homeobox domains do not appear to evolve asymmetrically in any gene duplicate pair (FET P-value of underrepresentation = 0.005). This 60 aa domain is highly conserved across distant species from insects to mammals [20]. However, it was reported to have incurred synonymous substitutions at reasonably high frequencies [21]. This suggests that functional divergence of newly created homedomain proteins does not involve changes to DNA binding specificity, but may instead be affected via changes in other, interaction/activation domains. Overall, such variability in the incidence of asymmetry within specific domains may prove informative in tracing functional divergence pathways.

### Asymmetrically evolving domains contain functionally important substitutions

Next, we analyzed the substitutions between two asymmetrically evolving copies of a domain to test whether such substitutions are more likely to contribute to functional differences between the two gene copies. We used the BLOSUM62 substitution matrix [22] to get the score for each substitution between the two copies. Lesser the score, rarer is the substitution and thus is more likely to impact function. We compared the asymmetrically evolving domains with those not deemed to be evolving asymmetrically in terms of average scores of the substituted residues between the two copies. We found that the substitution scores for the asymmetrically evolving domains were significantly lower compared to those for symmetrically evolving domains (Wilcoxon test P-value ranged from 0.04 to 2.9e-05 for the five fish species). Pooling all five fish species data yields a P-value of 2.3e-10. Interestingly, even when we control the average substitution scores by ensuring that for each asymmetrically evolving domain, we only sample a symmetrically evolving domain with average substitution score within 10 percentile using the pooled dataset, we still see a significant enrichment of low scores in the asymmetrically evolving pairs (Wilcoxon test P-value = 0.0005). This may suggest that for the same total evolution, the asymmetrically evolving domains contain functionally more consequential substitutions. However, we cannot rule out the possibility that the rarer substitutions arise due to increased mutation rates in the asymmetrically evolving copy.

### Asymmetrically evolving gene duplicates exhibit greater expression divergence

**Figure 3:**
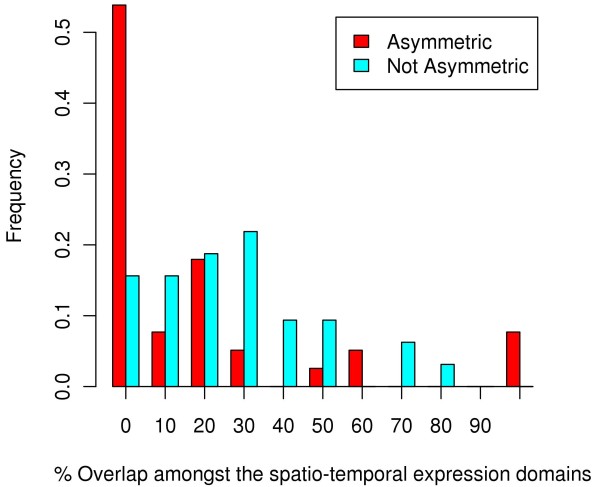
**Distribution of the normalized overlap between the spatio-temporal expression domains for asymmetrically and non-asymmetrically evolving*D. rerio*gene duplicates computed separately at every developmental stage.**

Having identified the duplicated genes that show significant asymmetry in their evolutionary rates and having found that the asymmetric pairs carry substitutions likely to have a greater functional impact, we next sought to determine whether this is accompanied by differences in spatio-temporal expression of the two copies. ZFIN database [10] provides comprehensive resource for *DR* including mRNA in situ hybridization and RT-PCR expression data. Of the 62 pairs of *DR* duplicate genes in our analysis that showed asymmetry (considering both multi-domain as well as single domain proteins) with DSA, 14 had expression localization data for at least one developmental stage in ZFIN for both copies. For the remaining 57 pairs that did not show asymmetry in any of their domains, 11 had expression localization data for at least one developmental stage in ZFIN for both copies. We computed for each such pair, the overlap (see Methods) between the expression domains. We found the asymmetrically evolving duplicates to have significantly less overlap (Wilcoxon test P-value = 0.002) (Figure 3). A notable example is the *atf7a/b* paralogs. Gene *atf7b* is widely expressed during embryogenesis (similar to the human ortholog *Atf7*) whereas *atf7a* is restricted to the notochord during segmentation [23], consistent with functional specialization of this gene after duplication.

Thus, for the *DR* duplicate gene pairs with available spatio-temporal expression data, we observed a significantly greater expression divergence for the asymmetrically evolving gene pairs as compared to the non-asymmetrically evolving gene pairs which suggests a possible functional manifestation of the rate variation either due to *NF* or *SF,* as has been shown previously [24-27].

## Conclusions

In this study we have carried out a large scale analysis of asymmetric evolution of gene duplicates in five teleost fish genomes [7]. These gene duplicates were a result of a teleost specific WGD event ~305-450 MYA [8,9] and thus have evolved over an identical evolutionary time, providing a suitable dataset for a large scale study. We detected asymmetric evolution in 50-65 % (at 10 % FDR) of the gene pairs using Fisher Exact Test, greater than what has been previously reported. A large fraction of the asymmetry detected at the whole protein level can be attributed to a specific domain. Moreover, several cases of asymmetric evolution were uniquely detected in the domain-centric analysis probably due to a better signal to noise ratio. Our results suggest that the asymmetry in evolutionary rate is primarily localized within specific domains and this is further supported by the finding that non-synonymous changes between the two duplicates are clustered within few of its domains and not spread evenly across all the domains. Most of the domains that showed a signal for asymmetry also showed clustering of non-synonymous changes within them. Asymmetrically evolving domains are targeted for qualitatively different substitutions as compared to symmetrically evolving domains, consistent with a functional divergence between duplicated genes. Interestingly, for gene duplicate pairs that harbored multiple asymmetrically evolving domains, in most cases, all of the faster evolving domain copies belonged to the same paralog; this is consistent with *NF* as the prevalent mechanism underlying diversification of the gene duplicates, also suggested by some of the previous studies [3-5]. Lastly, we found that the protein pairs with asymmetrically evolving domains showed significantly greater divergence in gene expression as compared to the protein pairs with no asymmetrically evolving domain. Overall, our study provides novel insights into the divergence of gene duplicates by focusing on asymmetric evolution of individual functional domains of the protein.

## Methods

### Duplicate gene pairs

The paralogs in five teleost fish genomes that showed phylogenetic and syntenic support for origin by WGD were reported in [7]. We downloaded these duplicate gene pairs: *Danio rerio (DR)* (615), *Oryzias latipes (OL)* (672), *Gasterosteus aculeatus (GA)* (775), *Tetraodon nigroviridis (TN)* (650) and *Takifugu rubripes (TR)* (702). The counts within the parenthesis are the number of gene duplicate pairs for each species. For each fish gene duplicates, we downloaded the corresponding Mouse orthologs from Ensembl v56. The gene orthology prediction in Ensembl has been generated by a pipeline that uses maximum likelihood phylogenetic gene trees that are reconciled with their species tree and annotated to distinguish duplication or speciation events [28]. We only retained the duplicate pairs for which each gene in the pair had one and the same Mouse ortholog, yielding the following numbers of duplicate pairs: *DR* (407), *OL* (603), *GA* (692), *TN* (406) and *TR* (540).

### Protein domain structure

We identified the protein domain architecture using Pfam 24.0 [29]. We only considered the high quality Pfam-A entries that satisfied the gathering threshold (GT) cutoff. The GT cutoff is set by Pfam curators for each model and is chosen to minimize false positives. We retained only the gene duplicates that have identical protein domain architecture to each other and to the corresponding Mouse ortholog. We further divided the gene duplicates into single and multiple domain groups. Table 4 provides a summary of the retained gene pairs.

**Table 4:** Summary of gene duplicates in five teleost fish species

**Species**	**Same protein domain structure**				**Different protein domain structure**
	**Single domain**	**Multiple domains**	**Total**	**Total (dS > =0.2 & dS < =2)**	
*D. rerio*	109	184	293	119	114
*O. latipes*	173	247	420	144	183
*G. aculeatus*	189	295	484	159	208
*T. nigrovirdis*	167	111	278	64	128
*T. rubripes*	226	162	388	119	152

### Test for asymmetry

For each gene duplicate pair and the corresponding mouse ortholog, we aligned the amino acid sequences using T-Coffee [30] and then converted them to nucleotide sequence alignment to compute the evolutionary rates using the tree topology shown in Figure 1. We applied the *branch model*[31] using the *codeml* program of PAML package [12], by setting the variable *model = 1* which fits the *free-ratios model* to compute the rate of non-synonymous substitutions (dN), rate of synonymous substitutions (dS) and the *ω* ratio (dN/dS) separately for each branch of the tree. We only considered the duplicate pairs for which dS along each of the branches leading to the fish duplicate gene from their last common ancestor was reasonably large (dS1, dS2 > = 0.2) and at the same time not saturated (dS1,dS2 < = 2) (Table 4) (Figure 1).

We used two approaches to assess asymmetry in evolutionary rates of the gene duplicates. The first approach uses the Likelihood-ratio test (LRT) to compare the fit of two likelihood models: (a) by allowing the ω to vary among the two branches leading to the observed duplicated fish gene sequences from the last common ancestor (ω1 and ω2), as well as among other branches (*model = 1*), (b) by constraining the two branches leading to the fish gene duplicates from the last common ancestor to have the same *ω* value (ω1 = ω2), while allowing other branches to vary freely (*model = 2*). The LRT statistic is calculated as: 2* |ln L1 – ln L2|, where L1 is the likelihood of the first model and L2 is the likelihood of the second model. We estimated the significance of improvement of fit by the first model over the second model using the chi-square test with one degree of freedom. We refer to this test as the LRT.

The second approach to test asymmetry was based on Fisher Exact test. As described above using the *free-ratios model* of codeml, we first estimated the number of synonymous (S) and non-synonymous (N) substitutions along the branches leading to the fish gene duplicates from their last common ancestor. By taking the synonymous substitutions as the background, we then compared the non-synonymous substitution between the two branches using Fisher Exact test. We refer to this test as FET. Given a 2x2 contingency table, FET essentially compute the fraction of all configurations of the contingency table that have a more extreme difference in the ratio of non-synonymous changes and the ratio of synonymous changes in the two branches.

For testing asymmetry using the *Domain Specific Analysis* and *Combined Domain Analysis* (see Results), we carried out the FET as described above except that we used the non-synonymous substitutions in the domain of interest as the foreground and the synonymous substitutions of the whole protein as the background.

### Assessing the false positive rate of FET approach to detect of asymmetric evolution

We estimated the branch lengths of the prototypical tree in Figure 1 from the actual data and used it to simulate evolution and assess false positive rate of our FET approach. The phylogenetic trees for Mouse and the duplicated genes of each of the five fish species: *DR, GA, OL, TN* and *TR* were estimated by running *DNAMLK* program of the PHYLIP package (http://www.phylip.com/) on the concatenated nucleotide sequences corresponding to all the orthologous Mouse proteins and two concatenated sequences corresponding to the two copies of the duplicated genes of the fish species. Based on synteny and/or phylogenetic information the fish duplicates were designated as copy-A and copy-B in [7] to indicate that one is the original ancestral copy while the other is created by WGD. We concatenated all ‘A’ copies together and all ‘B’ copies together. However, changing this grouping has no impact on our results. The branch lengths of the prototypical tree were estimated by averaging the corresponding branch lengths of these five trees. The resulting tree was then used as input to the evolver program of the PAML package [12] to simulate sequences of length 10,000 codons using the codon substitution model under neutral evolution (ω = 1) and all branches evolving at the same rate. Although the duplicated protein sequences were ~500 amino acid long on average, our use of the much longer sequences for simulation allows a very stringent estimation of the false positive rate. The simulation was carried out 1000 times. The generated sequences corresponding to the duplicated nodes were analyzed by the FET approach for asymmetric evolution as described previously.

### Clustering of non-synonymous changes within domains

For each duplicated protein with multiple domains, we computed N and S for every domain using *free-ratios model* of codeml as described above. We then used FET to compare N for a given domain with the sum of N for all the other domains of the protein, while using S in the domain and sum of S in other domains as the background, with the null hypothesis that every domain in the protein is an unbiased target of non-synonymous changes.

### Spatio-temporal expression divergence analysis

We obtained all available spatio-temporal expression data for *DR* genes from the ZFIN database [10]. For each *DR* gene, we compiled all the stages of development for which expression data were available, along with the anatomical regions where expression was observed at any particular developmental stage. We excluded any stage which showed ubiquitous expression of the gene in the whole organism. We refer to these anatomical regions as expression domains. For all *DR* gene duplicate pairs that were detected as evolving asymmetrically by our *Domain Specific Analysis* (see Results), separately at each developmental stage, we computed the ratio of intersection and union of expression domains for the two paralogs. We repeated this analysis for all the non-asymmetrically evolving gene duplicates.

## Abbreviations

WGD, Whole genome duplication; NF, Neofunctionalization; SF, Subfunctionalization; FET, Fisher’s exact test; LRT, Likelihood-ratio test.

## Authors’ contributions

SH conceived the study. SH and MK designed the experiments. MK carried out the experiments and analyzed the data. MK and SH wrote the paper. Both authors read and approved the final manuscript.

## Supplementary Material

Additional file 1 **Table S1.** Number and percentage of paralogs deemed to be asymmetrically evolving (FDR = 5 %) based on the whole protein and using Fisher Exact test (FET). **Table S2.** Number and percentage of paralogs deemed to be asymmetrically evolving (FDR = 0.01 %) based on the whole protein and using Fisher Exact test (FET). **Table S3.** Fisher exact test based analysis of asymmetrically evolving duplicate gene pairs using sampled codons to create artificial domains. **Table S4.** Number and percentage of paralogs deemed to be asymmetrically evolving (FDR = 10 %) based on the non-domain linker regions using Fisher Exact test (FET). **Table S5.** Duplicate gene pairs that contained multiple asymmetrically evolving domains categorized based on whether all the faster domains were in the same copy *(Category 1)* or distributed between the two copies *(Category 2)*. **Table S6.** Frequency of occurrence of each of the protein domains and the fraction of times they were detected to be evolving asymmetrically (FET P-value < = 0.05, FDR < = 20 %). **Supplementary Results.** Differing regions of the gene duplicates are targeted for non-synonymous substitutions. Click here for file
