# Supplementary material for: Functional divergence of gene duplicates – a domain-centric view
Source: BMC Evol Biol. 2012 Jul 27;12:126. doi: 10.1186/1471-2148-12-126 (PMC3426493; doi:10.1186/1471-2148-12-126)
Supplement: Additional file 1 — Table S1. Number and percentage of paralogs deemed to be asymmetrically evolving (FDR = 5 %) based on the whole protein and using Fisher Exact test (FET). Table S2. Number and percentage of paralogs deemed to be asymmetrically evolving (FDR = 0.01 %) based on the whole protein and using Fisher Exact test (FET). Table S3. Fisher exact test based analysis of asymmetrically evolving duplicate gene pairs using sampled codons to create artificial domains. Table S4. Number and percentage of paralogs deemed to be asymmetrically evolving (FDR = 10 %) based on the non-domain linker regions using Fisher Exact test (FET). Table S5. Duplicate gene pairs that contained multiple asymmetrically evolving domains categorized based on whether all the faster domains were in the same copy (Category 1) or distributed between the two copies (Category 2). Table S6. Frequency of occurrence of each of the protein domains and the fraction of times they were detected to be evolving asymmetrically (FET P-value < = 0.05, FDR < = 20 %). Supplementary Results. Differing regions of the gene duplicates are targeted for non-synonymous substitutions. [file 1471-2148-12-126-S1.docx]

**Supplementary Material**

**Supplementary Tables**

**Table S1.** Number and percentage of paralogs deemed to be asymmetrically evolving (FDR = 5%) based on the whole protein and using Fisher Exact test (FET).

| **Species** | **Asymmetry (FET)** |
| --- | --- |
| *D. rerio* | 77/119 (64.7%) |
| *O. latipes* | 86/144 (59.7%) |
| *G. aculeatus* | 80/159 (50.3%) |
| *T. nigrovirdis* | 35/64 (54.6%) |
| *T. rubripes* | 69/119 (57.9%) |

**Table S2.** Number and percentage of paralogs deemed to be asymmetrically evolving (FDR = 0.01%) based on the whole protein and using Fisher Exact test (FET).

| **Species** | **Asymmetry (FET)** |
| --- | --- |
| *D. rerio* | 33/119 (27.7%) |
| *O. latipes* | 41/144 (28.5%) |
| *G. aculeatus* | 38/159 (23.8%) |
| *T. nigrovirdis* | 18/64 (28.1%) |
| *T. rubripes* | 27/119 (22.7%) |

**Table S3.** Fisher exact test based analysis of asymmetrically evolving duplicate gene pairs using sampled codons to create artificial domains.

| **Species** | **Sampled CDA** | **Sampled DSA** | |
| --- | --- | --- | --- |
|  |  | wrt proteins | wrt domains |
| *D. rerio* | 25/45 (55.5%) | 21/45 (46.6%) | 32/134 (23.8%) |
| *O. latipes* | 35/67 (52.2%) | 31/67 (46.2%) | 52/209 (24.8%) |
| *G. aculeatus* | 34/68 (50%) | 27/68 (39.7%) | 43/209 (20.6%) |
| *T. nigrovirdis* | 10/25 (40%) | 16/25 (64%) | 19/67 (28.3%) |
| *T. rubripes* | 25/54 (46.3%) | 18/54 (33.3%) | 32/148 (21.6%) |

**Table S4.** Number and percentage of paralogs deemed to be asymmetrically evolving (FDR = 10%) based on the non-domain linker regions using Fisher Exact test (FET).

| **Species** | **Asymmetry (FET)** |
| --- | --- |
| *D. rerio* | 74/119 (62.2%) |
| *O. latipes* | 78/144 (54.2%) |
| *G. aculeatus* | 81/159 (50.9%) |
| *T. nigrovirdis* | 38/64 (59.4%) |
| *T. rubripes* | 50/119 (42.1%) |

**Table S5.** Duplicate gene pairs that contained multiple asymmetrically evolving domains categorized based on whether all the faster domains were in the same copy *(Category 1)* or distributed between the two copies *(Category 2)*

| **Species** | **Copy 1** | **Copy 2** | **Category** |
| --- | --- | --- | --- |
| *D. rerio* | ENSDARG00000043806 | ENSDARG00000061219 | 1 |
| *D. rerio* | ENSDARG00000052789 | ENSDARG00000035869 | 1 |
| *D. rerio* | ENSDARG00000005350 | ENSDARG00000016348 | 1 |
| *D. rerio* | ENSDARG00000024827 | ENSDARG00000009524 | 1 |
| *D. rerio* | ENSDARG00000018399 | ENSDARG00000058230 | 1 |
| *D. rerio* | ENSDARG00000007788 | ENSDARG00000012684 | 1 |
| *D. rerio* | ENSDARG00000043213 | ENSDARG00000042540 | 1 |
| *D. rerio* | ENSDARG00000041141 | ENSDARG00000053875 | 1 |
| *D. rerio* | ENSDARG00000070316 | ENSDARG00000018130 | 1 |
| *D. rerio* | ENSDARG00000051913 | ENSDARG00000058695 | 1 |
| *D. rerio* | ENSDARG00000002642 | ENSDARG00000067958 | 1 |
| *D. rerio* | ENSDARG00000033733 | ENSDARG00000022531 | 2 |
| *O. latipes* | ENSORLG00000003624 | ENSORLG00000010705 | 1 |
| *O. latipes* | ENSORLG00000012347 | ENSORLG00000017617 | 1 |
| *O. latipes* | ENSORLG00000009199 | ENSORLG00000006815 | 1 |
| *O. latipes* | ENSORLG00000008088 | ENSORLG00000002546 | 1 |
| *O. latipes* | ENSORLG00000012482 | ENSORLG00000001848 | 1 |
| *O. latipes* | ENSORLG00000008215 | ENSORLG00000005304 | 1 |
| *O. latipes* | ENSORLG00000007475 | ENSORLG00000015371 | 1 |
| *O. latipes* | ENSORLG00000006887 | ENSORLG00000019036 | 1 |
| *O. latipes* | ENSORLG00000008893 | ENSORLG00000001945 | 1 |
| *O. latipes* | ENSORLG00000003934 | ENSORLG00000016783 | 2 |
| *O. latipes* | ENSORLG00000001466 | ENSORLG00000016922 | 2 |
| *O. latipes* | ENSORLG00000005701 | ENSORLG00000004390 | 2 |
| *O. latipes* | ENSORLG00000000669 | ENSORLG00000012892 | 2 |
| *G. aculeatus* | ENSGACG00000012708 | ENSGACG00000003489 | 1 |
| *G. aculeatus* | ENSGACG00000013801 | ENSGACG00000019909 | 1 |
| *G. aculeatus* | ENSGACG00000008560 | ENSGACG00000017144 | 1 |
| *G. aculeatus* | ENSGACG00000001584 | ENSGACG00000001700 | 2 |
| *T. nigroviridis* | ENSTNIG00000005306 | ENSTNIG00000009173 | 1 |
| *T. nigroviridis* | ENSTNIG00000015850 | ENSTNIG00000009107 | 1 |
| *T. rubripes* | ENSTRUG00000012243 | ENSTRUG00000005544 | 1 |
| *T. rubripes* | ENSTRUG00000014771 | ENSTRUG00000016863 | 1 |
| *T. rubripes* | ENSTRUG00000012868 | ENSTRUG00000004558 | 1 |
| *T. rubripes* | ENSTRUG00000002332 | ENSTRUG00000011041 | 1 |

**Table S6.** Frequency of occurrence of each of the protein domains and the fraction of times they were detected to be evolving asymmetrically (FET P-value <= 0.05, FDR <= 20%).

| **Domain** | **Total count** | **Percent asymmetric** |
| --- | --- | --- |
| MARVEL | 5 | 100 |
| CSD | 3 | 100 |
| GDA1_CD39 | 3 | 100 |
| Glyco_transf_64 | 3 | 100 |
| Na_H_Exchanger | 3 | 100 |
| adh_short | 2 | 100 |
| Aldo_ket_red | 2 | 100 |
| ATP_Ca_trans_C | 2 | 100 |
| Band_7 | 2 | 100 |
| Caprin-1_C | 2 | 100 |
| CRM1_C | 2 | 100 |
| DUF3528 | 2 | 100 |
| Glyco_transf_29 | 2 | 100 |
| MBOAT | 2 | 100 |
| Ndr | 2 | 100 |
| NTR | 2 | 100 |
| P2X_receptor | 2 | 100 |
| PDEase_I | 2 | 100 |
| Sema | 2 | 100 |
| Somatomedin_B | 2 | 100 |
| Sulfotransfer_1 | 2 | 100 |
| Trypsin | 2 | 100 |
| zf-RanBP | 2 | 100 |
| zf-UBR | 2 | 100 |
| Aa_trans | 1 | 100 |
| ABC_membrane_2 | 1 | 100 |
| ADIP | 1 | 100 |
| Arf | 1 | 100 |
| Axin_b-cat_bind | 1 | 100 |
| Calsarcin | 1 | 100 |
| CH | 1 | 100 |
| Choline_transpo | 1 | 100 |
| COesterase | 1 | 100 |
| CRF-BP | 1 | 100 |
| DIX | 1 | 100 |
| DUF1977 | 1 | 100 |
| DUF3371 | 1 | 100 |
| ERbeta_N | 1 | 100 |
| ERM | 1 | 100 |
| FERM_M | 1 | 100 |
| FERM_N | 1 | 100 |
| FH2 | 1 | 100 |
| Fibrinogen_C | 1 | 100 |
| GAS2 | 1 | 100 |
| GluR_Homer-bdg | 1 | 100 |
| Hamartin | 1 | 100 |
| Hint | 1 | 100 |
| HJURP_C | 1 | 100 |
| Jun | 1 | 100 |
| Lgl_C | 1 | 100 |
| L_HGMIC_fpl | 1 | 100 |
| LLGL | 1 | 100 |
| LMBR1 | 1 | 100 |
| Metallophos | 1 | 100 |
| Molybdopterin | 1 | 100 |
| MOZ_SAS | 1 | 100 |
| Myosin_tail_1 | 1 | 100 |
| P16-Arc | 1 | 100 |
| PA | 1 | 100 |
| PG_binding_1 | 1 | 100 |
| PI-PLC-Y | 1 | 100 |
| PRK | 1 | 100 |
| Ricin_B_lectin | 1 | 100 |
| Sds3 | 1 | 100 |
| Sulfotransfer_2 | 1 | 100 |
| TF_Otx | 1 | 100 |
| TIMP | 1 | 100 |
| TRAM_LAG1_CLN8 | 1 | 100 |
| TRP_2 | 1 | 100 |
| Tweety | 1 | 100 |
| Eeig1 | 6 | 83 |
| Aminotran_5 | 5 | 80 |
| Myelin_PLP | 5 | 80 |
| LIM_bind | 4 | 75 |
| Pep_M12B_propep | 4 | 75 |
| UDPGP | 4 | 75 |
| Cyclin_N | 9 | 67 |
| SNF | 9 | 67 |
| Crystall | 6 | 67 |
| Pkinase_Tyr | 6 | 67 |
| Abhydrolase_1 | 3 | 67 |
| Amidohydro_1 | 3 | 67 |
| Ank | 3 | 67 |
| DUF1041 | 3 | 67 |
| Dymeclin | 3 | 67 |
| Integrin_B_tail | 3 | 67 |
| Orn_Arg_deC_N | 3 | 67 |
| Orn_DAP_Arg_deC | 3 | 67 |
| PAX | 3 | 67 |
| PID | 3 | 67 |
| RhoGEF | 3 | 67 |
| V-set | 3 | 67 |
| PMP22_Claudin | 11 | 64 |
| AMP-binding | 10 | 60 |
| Glycolytic | 5 | 60 |
| Neur_chan_memb | 5 | 60 |
| Oxysterol_BP | 5 | 60 |
| RGS | 5 | 60 |
| Pkinase | 26 | 50 |
| Dynamin_N | 6 | 50 |
| Gelsolin | 6 | 50 |
| MFS_1 | 6 | 50 |
| Tetraspannin | 6 | 50 |
| Acyl-CoA_dh_N | 2 | 50 |
| Anoctamin | 2 | 50 |
| BAR | 2 | 50 |
| Collagen | 2 | 50 |
| Cyclin_C | 2 | 50 |
| DUF2370 | 2 | 50 |
| DUF747 | 2 | 50 |
| Dynamin_M | 2 | 50 |
| Ephrin | 2 | 50 |
| EXS | 2 | 50 |
| Gastrin | 2 | 50 |
| GED | 2 | 50 |
| Guanylate_cyc | 2 | 50 |
| Hormone_2 | 2 | 50 |
| IGFBP | 2 | 50 |
| IML2 | 2 | 50 |
| IP_trans | 2 | 50 |
| KH_1 | 2 | 50 |
| Laminin_N | 2 | 50 |
| Myosin_head | 2 | 50 |
| NIF | 2 | 50 |
| PBD | 2 | 50 |
| Peptidase_C2 | 2 | 50 |
| PIP49_C | 2 | 50 |
| PKK | 2 | 50 |
| Porin_3 | 2 | 50 |
| T-box | 2 | 50 |
| TEA | 2 | 50 |
| TGFb_propeptide | 2 | 50 |
| Thyroglobulin_1 | 2 | 50 |
| UPF0005 | 2 | 50 |
| wnt | 2 | 50 |
| zf-C3HC4 | 2 | 50 |
| A_deaminase | 4 | 50 |
| DCX | 4 | 50 |
| Disintegrin | 4 | 50 |
| DMAP_binding | 4 | 50 |
| DnaJ | 4 | 50 |
| ELFV_dehydrog | 4 | 50 |
| Fasciclin | 4 | 50 |
| HABP4_PAI-RBP1 | 4 | 50 |
| HCO3_cotransp | 4 | 50 |
| MH1 | 4 | 50 |
| NAD_binding_2 | 4 | 50 |
| PAP2 | 4 | 50 |
| RA | 4 | 50 |
| Sugar_tr | 4 | 50 |
| E1-E2_ATPase | 5 | 40 |
| Ldh_1_C | 5 | 40 |
| Macoilin | 5 | 40 |
| 7tm_1 | 6 | 33 |
| ABC2_membrane | 3 | 33 |
| Arfaptin | 3 | 33 |
| ASF1_hist_chap | 3 | 33 |
| bZIP_1 | 3 | 33 |
| C2 | 9 | 33 |
| CBS | 6 | 33 |
| EF_assoc_2 | 3 | 33 |
| F5_F8_type_C | 3 | 33 |
| Hemopexin | 12 | 33 |
| LIM | 18 | 33 |
| Miro | 3 | 33 |
| PGAM | 6 | 33 |
| Pyridoxal_deC | 3 | 33 |
| TLE_N | 3 | 33 |
| UQ_con | 3 | 33 |
| V_ATPase_I | 3 | 33 |
| Y_phosphatase | 6 | 33 |
| I-set | 16 | 31 |
| SH3_1 | 10 | 30 |
| ABC_tran | 4 | 25 |
| Band_3_cyto | 4 | 25 |
| CRAL_TRIO_N | 4 | 25 |
| ELFV_dehydrog_N | 4 | 25 |
| MH2 | 4 | 25 |
| PIP5K | 4 | 25 |
| Pkinase_C | 4 | 25 |
| Reprolysin | 4 | 25 |
| SRCR | 4 | 25 |
| zf-C2H2 | 4 | 25 |
| EGF_2 | 9 | 22 |
| Ras | 9 | 22 |
| PH | 14 | 21 |
| Annexin | 20 | 20 |
| EGF | 5 | 20 |
| Hydrolase | 5 | 20 |
| Neur_chan_LBD | 5 | 20 |
| TIG | 7 | 14 |
| Ion_trans | 8 | 13 |
| Mito_carr | 12 | 8 |
| fn3 | 13 | 8 |
| WD40 | 52 | 2 |
| Homeobox | 19 | 0 |
| RRM_1 | 14 | 0 |
| HLH | 7 | 0 |
| MORN | 8 | 0 |
| FGF | 6 | 0 |
| Laminin_EGF | 6 | 0 |
| OAR | 6 | 0 |
| Cation_ATPase_C | 5 | 0 |
| Cation_ATPase_N | 5 | 0 |
| Ldh_1_N | 5 | 0 |
| CRAL_TRIO | 4 | 0 |
| Erf4 | 4 | 0 |
| GoLoco | 4 | 0 |
| LisH | 4 | 0 |
| PFK | 4 | 0 |
| RUN | 4 | 0 |
| WH2 | 4 | 0 |
| zf-A20 | 4 | 0 |
| zf-AN1 | 4 | 0 |
| zf-C2H2_jaz | 4 | 0 |
| zf-MIZ | 4 | 0 |
| 4HBT | 2 | 0 |
| 7tm_3 | 1 | 0 |
| Abi_HHR | 2 | 0 |
| Acyl-CoA_dh_1 | 2 | 0 |
| Acyl-CoA_dh_M | 2 | 0 |
| AFG1_ATPase | 1 | 0 |
| ANF_receptor | 1 | 0 |
| ANTH | 1 | 0 |
| ArfGap | 1 | 0 |
| Arrestin_C | 3 | 0 |
| Arrestin_N | 3 | 0 |
| ATP-grasp_2 | 3 | 0 |
| B56 | 1 | 0 |
| BTG | 3 | 0 |
| C1_1 | 2 | 0 |
| C1q | 2 | 0 |
| Ca_chan_IQ | 1 | 0 |
| Cadherin | 1 | 0 |
| Calpain_III | 2 | 0 |
| Calreticulin | 1 | 0 |
| CaMBD | 1 | 0 |
| CBFNT | 1 | 0 |
| CDC50 | 1 | 0 |
| ChaC | 1 | 0 |
| Citrate_synt | 3 | 0 |
| CNH | 2 | 0 |
| cNMP_binding | 2 | 0 |
| CoA_binding | 3 | 0 |
| Copine | 1 | 0 |
| Cullin | 2 | 0 |
| Cullin_Nedd8 | 2 | 0 |
| CUT | 1 | 0 |
| DAGK_acc | 1 | 0 |
| DAGK_cat | 1 | 0 |
| Ded_cyto | 2 | 0 |
| DFDF | 2 | 0 |
| DNA_photolyase | 3 | 0 |
| Drf_FH3 | 1 | 0 |
| Drf_GBD | 1 | 0 |
| DSL | 1 | 0 |
| DUF1899 | 2 | 0 |
| DUF1900 | 2 | 0 |
| DUF1982 | 1 | 0 |
| DUF298 | 1 | 0 |
| DUF300 | 2 | 0 |
| DUF3377 | 1 | 0 |
| DUF3395 | 2 | 0 |
| DUF3398 | 2 | 0 |
| DUF3694 | 2 | 0 |
| E2_bind | 1 | 0 |
| EF_assoc_1 | 3 | 0 |
| efhand_like | 1 | 0 |
| Engrail_1_C_sig | 1 | 0 |
| Enolase_C | 2 | 0 |
| Enolase_N | 2 | 0 |
| ENTH | 1 | 0 |
| Exostosin | 3 | 0 |
| FAD_binding_7 | 3 | 0 |
| FCH | 3 | 0 |
| Fer2 | 1 | 0 |
| FERM_C | 1 | 0 |
| FFD_TFG | 2 | 0 |
| FHA | 2 | 0 |
| Fork_head | 2 | 0 |
| Furin-like | 1 | 0 |
| FYVE | 2 | 0 |
| GAT | 1 | 0 |
| Glyco_hydro_1 | 1 | 0 |
| Glycos_transf_2 | 1 | 0 |
| Gtr1_RagA | 1 | 0 |
| HH_signal | 1 | 0 |
| Hormone_recep | 2 | 0 |
| IBN_N | 2 | 0 |
| IBR | 2 | 0 |
| IMD | 2 | 0 |
| Integrin_b_cyt | 3 | 0 |
| Integrin_beta | 3 | 0 |
| Ion_trans_2 | 1 | 0 |
| IQ | 1 | 0 |
| JmjC | 1 | 0 |
| KIF1B | 2 | 0 |
| Kinesin | 2 | 0 |
| K_tetra | 3 | 0 |
| Ligase_CoA | 3 | 0 |
| Lipin_N | 1 | 0 |
| LNS2 | 1 | 0 |
| Lysyl_oxidase | 1 | 0 |
| Med26 | 2 | 0 |
| MIT | 1 | 0 |
| MNNL | 1 | 0 |
| Mtp | 2 | 0 |
| Myosin_N | 1 | 0 |
| NADH-G_4Fe-4S_3 | 1 | 0 |
| NCD3G | 1 | 0 |
| nlz1 | 3 | 0 |
| NOT2_3_5 | 2 | 0 |
| Not3 | 2 | 0 |
| OLF | 1 | 0 |
| Orai-1 | 2 | 0 |
| OSR1_C | 1 | 0 |
| OTU | 2 | 0 |
| PAE | 1 | 0 |
| PAS | 2 | 0 |
| Pax7 | 3 | 0 |
| PDZ | 3 | 0 |
| Peptidase_C14 | 1 | 0 |
| Peptidase_M10 | 1 | 0 |
| Peptidase_M24 | 1 | 0 |
| Phosducin | 1 | 0 |
| PI-PLC-X | 1 | 0 |
| PrmA | 1 | 0 |
| Proteasome | 1 | 0 |
| Proteasome_A_N | 1 | 0 |
| PSI | 2 | 0 |
| PTB | 1 | 0 |
| PX | 3 | 0 |
| RanBPM_CRA | 2 | 0 |
| Recep_L_domain | 2 | 0 |
| Ribosomal_L7Ae | 1 | 0 |
| RPE65 | 1 | 0 |
| SDF | 2 | 0 |
| Senescence | 1 | 0 |
| SH2 | 2 | 0 |
| SH3_2 | 2 | 0 |
| SK_channel | 1 | 0 |
| SPX | 2 | 0 |
| SRF-TF | 1 | 0 |
| START | 1 | 0 |
| Stathmin | 3 | 0 |
| Steroid_dh | 1 | 0 |
| Synaptobrevin | 1 | 0 |
| TGF_beta | 2 | 0 |
| ThiF | 1 | 0 |
| Tim44 | 1 | 0 |
| TPR_1 | 3 | 0 |
| Tyrosinase | 1 | 0 |
| UBA | 3 | 0 |
| UBACT | 1 | 0 |
| UBA_e1_thiolCys | 1 | 0 |
| VHP | 2 | 0 |
| VHS | 1 | 0 |
| WH1 | 2 | 0 |
| WW | 1 | 0 |
| WWE | 1 | 0 |
| Xpo1 | 2 | 0 |
| zf-B_box | 1 | 0 |
| zf-C2HC | 1 | 0 |
| zf-C4 | 2 | 0 |
| zf-DHHC | 2 | 0 |

**Supplementary Results**

**Differing regions of the gene duplicates are targeted for non-synonymous substitutions**

Given the mouse ortholog and the two fish paralogs, we identified the sites in the mouse protein that were mutated in exactly one of the two fish paralogs. Let, M1 represent the set of sites (positions) uniquely substituted in the first fish paralog, and let M2 represent the set of sites uniquely substituted in the second fish paralog. We tested whether the positions in M1 and M2 were interleaved or formed distinct contiguous clusters. To do so, we compared the inter-position distances within M1, within M2, and between M1 and M2. We found that the within-M1 and within-M2 distances were significantly smaller than the between-M1-M2 distances (Wilcoxon P-value < 3.4e-15). Thus the unique mutations in either of the copies lie closer to one another than they do to the unique mutations in the other copy which suggest that different regions of the gene duplicates are targeted for mutations.
